# The comparison of anxiety and depression levels in asthma and COPD patients

**DOI:** 10.1186/1939-4551-8-S1-A51

**Published:** 2015-04-08

**Authors:** Aysenaz Özcan, Adile Berna Dursun, Tugba Cicek

**Affiliations:** 1 Ataturk Chest Diseases and Thoracic Surgery Training and Research Hospital, Turkey; 2 Recep Tayyip Erdogan University School of Medicine, Turkey

## Background

Both asthma and COPD affect mental health due to their impact on activities, sleep and social life of patients and can be resulted in anxiety and depression. The objective of the study was to determine and compare the prevalence of anxiety and depression in asthma and COPD patients.

## Methods

Subjects without known psychiatric diseases were consecutively recruited from pulmonary and allergy out-patient clinics at the third level hospitals. Diagnosis of asthma and COPD was based on GINA and GOLD guideline, respectively. Depression and anxiety symptoms were evaluated using Beck Depression Inventory (BDI) and Beck Anxiety Inventory (BAI).

## Results

Study group consisted of 53 patients-30 with asthma and 22 with COPD. All patients had moderate-to severe diseases. COPD group was older, had more smoking history and higher number of hospitalization than those in asthmatics. Atopy rate and education level was higher in asthmatics than those in COPD group. The mean BDI and BAI scores were 17.96±12.39 and 20.57± 12.67 in the whole group. Both group had similar degree of anxiety (21.23±11.7 vs. 19.7±14), whereas BDI score was significantly higher in COPD group than that in asthmatics (15.63±13.6 vs. 21±10.1) (p=.02). Age, gender, smoking history, education level, atopy status, number of emergency room admission was not correlated with scores of BDI and BAI for both group. The only effective parameter on BDI score in COPD patients was having comorbidities

## Conclusions

Both asthmatics and COPD patients regardless of their sociodemographic and clinical features have similar degree of anxiety. In addition COPD patients are more depressive than asthmatics. The study indicates that psychiatric evaluation should be a part of tailoring therapy in chronic respiratory diseases.

